# Editorial: Highlights in contraception and family planning 2021/22

**DOI:** 10.3389/fgwh.2023.1238741

**Published:** 2023-09-05

**Authors:** Berna Dilbaz

**Affiliations:** Health Sciences University, Ankara, Türkiye

**Keywords:** contraception, family planning, fertility awareness, abortion, population-based

**Editorial on the Research Topic** Highlights in contraception and family planning 2021/22

We are very proud to have the chance to present several very interesting studies on contraception and family planning in this Research Topic. The research articles address a wide variety of subjects related to reproductive health, contraception, and family planning from very different countries with distinct settings. The Research Topic on *Highlights in Contraception and Family Planning 2021/22* constitutes 13 original articles written by 74 authors and has been well-received as there have been 24,000 views and 4,070 downloads.

The article titled *Identifying Client Targets for Improved Mobilization and Uptake of Integrated Family Planning and Reproductive Health in Environmental Programs in Kenya* is a Brief Research Report article (Obat et al.). As a part of a population health environment program, data were collected from clients by community-based distributors from four environmental community-based organisations, and the contraceptive preferences and demographic features of the population were analyzed. This article emphasises the partnership in conducting reproductive health programs.

Gutin et al. published a Review titled *“What if They Are Pre-conception? What Should We Do?”: Knowledge, Practices, and Preferences for Safer Conception Among Women Living With HIV and Healthcare Providers in Gaborone, Botswana*. They conducted qualitative in-depth interviews with 10 HIV healthcare providers and 10 WLHIV in Gaborone. Interviews were analyzed using a phenomenological approach. As safer conception knowledge was limited, the need for guidelines was pointed out.

Taylor et al. stated that unregulated sperm donations occur due to restrictive UK policies and practices for regulated donor insemination in their article *Are UK Policies and Practices for Regulated Donor Insemination Forcing Women to Find Unregulated Sperm Donors Online? A Perspective on the Available Evidence*. Alhassan and Madise examined the demand for modern family planning methods in urban Malawi and the needs of underserved groups in *Demand for Family Planning Satisfied With Modern Methods in Urban Malawi: CHAID Analysis to Identify Predictors and Women Underserved With Family Planning Services*.

Jonas et al. in their research article *Factors Associated With the Use of the Contraceptive Implant Among Women Attending a Primary Health Clinic in Cape Town, South Africa* analyzed the factors significantly associated with the intention of using the implant. A Brief Research Report by Wollum et al. summarised the results of the scales used for evaluating abortion experiences 6 months after abortion.

The Perspective article titled *The Potential of Self-Managed Abortion to Expand Abortion Access in Humanitarian Contexts* addresses the challenges that refugees and displaced people face in accessing abortion services. Delvaux et al. conducted a systematic review and narrative synthesis on the acceptability and satisfaction of contraceptive vaginal rings.

A qualitative study about women's perceptions of empowerment in relation to fertility intentions and family planning practices from Mozambique aimed to identify various factors that positively or negatively influence women's empowerment (Castro Lopes et al.). Halleran et al. aimed to analyze the knowledge of women who were trying to conceive about the timing and identification of the fertile window by using the Fertility Knowledge Questionnaire.

Ryan et al. reported the effects of three contraceptive methods (IUD, Depot-MPA, and levonorgestrel implant) on serum estradiol levels using data from the Evidence for Contraceptive Options and HIV Outcomes (ECHO) randomised trial. A qualitative study from Kenya investigated contraceptive method use trajectories among young women in Kenya and highlighted the needs of young women (Calhoun et al.). Finally, an original research article from Korhogo, Côte d'Ivoire, examined libido-sexual disorders and the abandonment of injectable contraceptives among users of the Ivorian Association for Family Well-Being (Essis et al.).

Family planning, reproductive health, and contraception are very important elements of well-being. Research in this area is required to improve service delivery and increase accessibility to modern family planning methods. We would like to thank the authors, editors, and reviewers for their precious scientific contributions.

